# Optimization of thermosonication conditions for critical quality parameters of watermelon juice using response surface methodology

**DOI:** 10.1038/s41598-024-64066-9

**Published:** 2024-06-14

**Authors:** Makaepea M. Maoto, Daniso Beswa, Afam I. O. Jideani

**Affiliations:** 1 Department of Food Science and Technology, Faculty of Science, Engineering and Agriculture, University of Venda, Private Bag 5050, Thohoyandou, Limpopo 0950 South Africa; 2 Department of Life and Consumer Sciences, School of Agriculture and Life Sciences, College of Agriculture and Environmental Sciences, University of South Africa, Private Bag x6, Florida, Gauteng 1710 South Africa; 3 Special Interest Group Post Harvest Handling, ISEKI-Food Association, Muthgasse 18, 1190 Vienna, Austria; 4 University of Johannesburg, Johnannesburg, South Africa

**Keywords:** Nutritional juice, Thermosonication, Quality parameters, Watermelon juice, Biochemistry, Biotechnology

## Abstract

Topical consumer interest in natural, healthier, safer and nutritional juice, has inspired the search for innovative technologies that can minimize product degradation. In this regard, thermosonication has been proposed as a potential processing technology that can preserve and produce “fresh” products. Watermelon (*Citrullus* *lanatus*) juice is a nutrient-rich fruit juice that is desired by consumers due to its appealing color, pleasant odor, sweet taste and low-calorie content. This fruit juice is, however, highly perishable and prone to microorganisms, because of its neutral pH value and high amount of water activity. In addition, it is thermo-sensitive and therefore degrades quickly under thermal processing. This study aimed to identify the optimal thermosonication processing conditions for retaining the critical quality parameters (lycopene, β-carotene, ascorbic acid and total polyphenolic content) of watermelon juice. Response surface methodology, employing a central composite design, was used to determine the effects of temperature (18–52 °C), processing time (2–13 min) and amplitude level (24–73 μm) at a constant frequency of 25 kHz. The highest quality parameters were obtained at 25 °C, 2 min, and 24 µm at a constant frequency of 25 kHz, which resulted in lycopene of 8.10 mg/100 g, β-carotene of 0.19 mg/100 g, ascorbic acid of 3.11 mg/100 g and total polyphenolic content of 23.96 mg/GAE/g with a desirability of 0.81. The proposed model was adequate (*p* < 0.0001), with a satisfactory determination coefficient (R^2^) of less than 0.8 for all phytochemicals. Thermosonicated watermelon juice samples showed minimal changes in their phytochemical properties, when compared to fresh juices; the lycopene content showed a significant increase after thermosonication, and a significant retention of β-carotene, ascorbic acid and total polyphenolic acid was observed. According to the findings, thermosonication could be a viable method for preserving watermelon juice, with minimal quality loss and improved functional attributes.

## Introduction

There has been an upsurge in interest in the consumption of natural fruit juices to substitute traditional caffeine-containing beverages such as coffee, tea or carbonated soft drinks^1^. This trend by health-conscious consumers has prompted the need to expand the supply of fruit juices^2^. Fruits are ideal ingredients for functional beverages. Considering their nutritional value, fruit juices can have functional properties such as antioxidant, antimicrobial, anti-inflammatory, and anti-cancer properties which are beneficial to human health^3^. Native to tropical Africa, the watermelon fruit (*Citrullus lanatus*) is a member of the *Cucurbitaceae* family^4^, preferred by consumers for its sweet, edible flesh and juice and its refreshing characteristics^5^. Its biomass is categorized into three components: flesh, seeds, and rind^6^. The flesh contributes to approximately 68% of the total weight, the rind approximately 30%, and the seeds about 2%^7^. Watermelon is considered the second most cultivated fruit in the world^4^, with a global production of almost 103 million tons in 2018^7^. In 2018/2019, the preliminary production volume of watermelons in South Africa amounted to 91.4 thousand metric tons, but in 2023 that market reduced slightly^8^.

Watermelon is traditionally consumed in its fresh form but in recent years several cuisines employ its pulp for the preparation of juices, jellies, ice cream and sweets in general^9^. Due to its high-water content, watermelon is mostly processed in to fruit juice to tame the summer thirst^10,11^. This nutritional fruit juice has a desirable color, pleasant odor and sweet taste, and can be a constituent of a healthy diet due to its nutritional content, low cholesterol, low saturated fat and low sodium levels^12^. It is rich in minerals, specific amino acids and phytochemicals, such as total polyphenolic content (TPC), β-carotene, ascorbic acid (AA) and lycopene^5^. Of all the fruits, watermelon contains the highest bioavailable lycopene, containing 60% more than heat-processed tomato juice^13^. Watermelon juice also contains phytochemicals which have antioxidant and anti-inflammatory properties^10^. Antioxidants prevent and repair the damage caused by free radicals to the human body, and reduce the risk of various diseases including hypertension, inflammatory illnesses, cancers, diabetes, and arthritis^14,15^. In addition, antioxidants combat oxidative stress and prevent the occurrence of cardiovascular disorders, ageing as well as neurodegenerative diseases^1^. Watermelon juice has been classified as a functional food due to its significant amounts of amino acid L-citrulline and electrolytes; as a result, it is recommended as a functional beverage aiding muscle recovery during exercise^10,16^. Therefore, inclusion of watermelon juice in a diet could help reducing degenerative human diseases; also reduce their negative side effects.

Watermelon juice is highly perishable, which makes it susceptible to microbial activity, because of its neutral pH value and high amount of water activity^13^. Santos et al.^17^ reported that, its shelf-life is approximately four days under room temperature. Low acidity and high-water content are what make the perishability of watermelon juice^18^. The perishable nature of watermelon juice creates a need for processing to increase its shelf-life and utilization^16^. Initially, the shelf life of watermelon juice was extended through the application of thermal pasteurization aimed at reducing the microbial and enzymatic load, but that deteriorated the quality of the juice^18^. This resulted in severe adverse effects in chemical reactions that led to a loss of color, the formation of novel compounds by the Maillard reaction, a reduction in nutrients, unpleasant sensory attributes and the degradation of phytochemicals in the beverage^10,19^. Because the watermelon fruit is thermo-sensitive^13^, watermelon-based products consequently tend to be limited in the market^6^. This state of affairs prompted researchers to search for alternative novel technology which can process watermelon juice without degrading its quality and health-promoting properties^20^. In line with this, thermosonication treatment, also known as ultrasonic-assisted heat treatment, is considered to be a smart and excellent innovative technological alternative to overcoming the thermal treatment limitations in processing fruit juices^4^. This innovative technique combines acoustic energy with moderate heat treatments to inactivate enzymes, pathogenic and spoilage microorganisms^21,22^. Thermosonication is an innovative approach which extends the shelf-life of foods and beverages, while retaining their nutritional, phytochemical compounds and enhancing their sensory qualities ^23^. The effectiveness of thermosonication is attributed to cell disruption caused by acoustic cavitation, which is determined by the frequency, power intensity and processing time^21,24^. Thermosonication induces the oxidative stress on fruits by utilizing the ultrasound to generate cavitation bubbles that release a massive amount of energy upon collapse, which effectively disintegrates water molecules into highly reactive ·OH radicals^4^. Yıkmıs et al.^25^, observed that thermosonication retained the bioactive compounds and minerals of black grape juice. Additionally, Zhang et al.^4^ reported that, thermosonication has induced oxidative stress of phenolics, leading to an increase in antioxidant capacity of fruit juice. Moreover, Kalsi et al.^22^ reported that thermosonication not only retained high content of ascorbic acid but also enhanced the levels of total phenols, flavonoids and antioxidant activity in guava juice. Additionally, thermosonication has proven to be effective in retaining the quality parameters of *kutkura* juice ^26^, apple juice^27^, and blueberry nectar^28^. The application of thermosonication thus has the potential to enable the processing of watermelon juice, with a high retention of desirable quality attributes. The desired quality attributes and increased shelf-life of watermelon juice may be achieved by optimizing its processing conditions using response surface methodology (RSM)—a model which employs mathematical and statistical techniques to analyse interactions between factors that influence experimental response values, it determines precise and optimal parameter combinations^29^. The model shows the influence of different independent variables and their interactions with the dependent variables under investigation, by creating a set of numerical models and producing a predictive equation, relating the values of the coefficients^30^. This study therefore aimed to determine the optimum thermosonication processing conditions for the preservation of selected phytochemicals in watermelon juice, using RSM.

## Materials and methods

### Sample collection and thermosonication

Three unbruised watermelons (*Sugar baby cv*) fruits per row were manually and randomly picked at the ripe stage from Valley Farms, South Africa. Their maturity was evident from the browning and drying of tendrils nearest the fruit, the loss of surface gloss, yellowing of the ground spot, and a dull instead of metallic sound produced when the fruit was thumped with the knuckles^15^. The watermelons weighed between 7.43 and 7.48 kg each. Two watermelon fruits per row were cut to ensure the validity of the methodology just before harvest. The fruits were transported to the Food Science laboratory at the Florida campus of the University of South Africa for processing. To remove soil residues, the watermelons were thoroughly washed under running tap water and kept overnight at about room temperature to simulate grocery store/retail conditions. The juice of each watermelon was extracted the following day. The watermelons were cut into 8–9 cm cubes, and the rind and seeds were separated from the flesh using a knife. The flesh was put in the table juice extractor (Russell Hobbs Juice Sensation Model no: RHJM01. 220–240 V–700 W, UK) to extract the juice. Next, the watermelon juice was thermosonicated using a combination of an ultrasound bath (Water bath, ST 30, Turkey) and a Sonicator probe (QSonica, Model Q700, USA). The juice was processed according to the generated independent variables at a constant frequency of 25 kHz (Table 1). Sixteen samples of treated fruit juice were placed in 50 ml polyethylene bottles and stored at − 20 °C for 72 h before analysis^13^. All reagents were supplied by Merck Chemical Suppliers.

**Table 1:** Optimization of thermosonication processing variables for watermelon juice.

Run	Independent variables		
	Temperature (°C)	Time (min)	Amplitude (µm)
1	25	10	24
2	45	2	24
3	35	6	42
4	25	10	61
5	45	10	61
6	25	2	61
7	35	6	73
8	45	10	24
9	35	6	43
10	25	2	24
11	35	6	11
12	35	6	42
13	45	2	61
14	52	6	42
15	35	13	42
16	18	6	42

(°C = degree Celsius; min = minutes; µm = micrometer).

### Experimental design

The experiment was done using RSM. Central composite design (CCD) was employed to study the combined effect of three independent variables—time, temperature, and amplitude^29^. Design Expert (statistical software version 6, Stat-ease Inc., Minneapolis MN, USA) was used. The CCD with a quadratic model was employed to produce the experimental designs, statistical analysis and regression model^30^. Amplitude (µm), temperature (°C) and processing time (min) were used as independent variables (Table 1). A total of 16 different combinations were generated, according to the CCD (Table 1). The dependent variables measured from the juice were lycopene, β-carotene, AA and TPC. Response surface plots which showed the values of the dependent variables were plotted using CCD.

### Determination of lycopene and β-carotene in watermelon juice

Lycopene and β-carotene were analyzed according to method by Olives-Barba et al.^31^ with some modification. In brief, aapproximately 3 g of watermelon juice was placed in a vessel. The watermelon juice was mixed with 100 ml of extraction solvent (methanol/Tetrahydrofuran (THF) (50:50 v/v) and hexane/acetone/ethanol (50:25:25 v/v/v) separately. The mixture was stirred for 30 min. The sample was then centrifuged at 1890 rpm for 15 min at 4 °C. The methanol/THF (50:50 v/v) extract was diluted to a concentration of THF lower than 10%; for the hexane/acetone/ethanol extract 15 ml of distilled water was added; the upper layer was placed in a round-bottom flask and an aliquot of 10 ml of the extract was evaporated to dryness under nitrogen flow under vacuum. The residues were dissolved to a final volume of 10 ml. The final solution was filtered through 0.45 µm membrane filters and 100 µL was injected for analysis using HPLC with a detector at absorbance 450 nm for β-carotene and 475 nm for lycopene. The mobile phase was a mixture of methanol and acetone (90/10 v/v) at a flow rate of 0.9 ml/ min. The column temperature was 30 °C. The sample was assayed by mobile phase of methanol/ACN + TEA 9 µM, the mobile phase was filtered through a 0.45 µm membrane and degassed ultrasonically prior to use. The absorbance was read at 450 nm for β-carotene and 475 nm for lycopene^31^.

### Determination of AA in watermelon juice

The ascorbic acid (AA) was determined by HPLC (Model RD-20A, Japan) using the method of Soliva-Fortuny et al.^32^. Approximately 25 ml of watermelon fruit juice was homogenised with 10 ml of extraction solution (10% metaphosphoric acid + 0.5% 2, 3 dimercapto-1-propanol, BAL, a thiol-reducing reagent). The homogenised mixture was centrifuged at 1890 rpm for 15 min at 4 °C. The supernatant was vacuum-filtered through Whatman No. 1 paper and diluted to 50 ml with distilled water. Then, samples were passed through a 0.45 µm membrane filter. An aliquot of 20 µl was injected into the HPLC system using an NH_2_ Spherisorb S5 column (250 4.6 mm, 5 µm). Detection was performed with a 486-absorbance detector at 254 nm.

### Determination of TPC in watermelon juice

TPC (expressed as gallic acid equivalent) was determined according to the method of Soliva-Fortuny et al.^32^ with slight modifications described by Shi et al.^11^. In brief, aapproximately 1.0 ml of watermelon juice was mixed with 1.0 ml of Na_2_CO_3_ solution (2 g/100 ml H_2_O) and kept at room temperature for 3 min. After the addition of 0.2 ml Folin-Ciocalteu reagent (twofold diluted with H_2_O), the reaction was kept for 30 min in the dark room, followed by centrifugation (Model 5702, RH, Japan) at 13.400 rpm for 5 min. The absorbance of supernatant was measured at 765 nm by using a UV-2600 UV/vis spectrophotometer (Shimadzu, Japan).

### Statistical analysis

The data analysis was performed using Design Expert statistical software version 6 (Stat-ease Inc., Minneapolis MN, USA)^30^. The analysis of variance (ANOVA) and regression coefficients of individual phytochemicals in terms of actual factors, were determined. The significances of all terms in the polynomial were statistically analyzed, at a probability level (p) of 0.0001, while the regression coefficient of determination (R^2^) was used to determine the adequacy of each model. The linear models were used to predict the responses. These values were related to the coded variables by a second-degree polynomial using the equations and plots.

## Results and discussion

### Effects of thermosonication on the lycopene content of the watermelon juice

Lycopene is a fat-soluble red-coloured phytochemical that has attracted significant interest since it is deemed to play a vital role in preventing chronic diseases such as cancer and diabetes^33,34^. The fresh watermelon juice recorded a lycopene content of 6.19 mg/100 g (Table 2) which was less than the 12.20 mg/100 g reported by Oberoi and Sogi^35^. The difference in these values may be due to agricultural practices and climate factors^14^.

**Table 2:** Effects of thermosonication processing variables on phytochemicals of watermelon juice.

Run	Independent variables			Dependent variables			
	Temperature	Time	Amplitude	Lycopene	β-carotene	AA	TPC
	(0 °C)	(min)	(µm)	mg/100 g	mg/100 g	mg/100 g	mg/GAE/g
Control	0	0	0	6.19	0.23	5.29	25.95
1	25	10	24	7.91	0.18	2.99	22.76
2	45	2	24	3.34	0.02	2.01	8.11
3	35	6	43	4.45	0.03	2.12	14.97
4	25	10	61	7.78	0.16	2.65	22.46
5	45	10	61	3.04	0.02	1.98	7.78
6	25	2	61	8.01	0.18	2.86	23.76
7	35	6	73	4.36	0.03	2.08	14.57
8	45	10	24	3.25	0.02	1.98	7.91
9	35	6	43	4.45	0.03	2.12	14.97
10	25	2	24	8.10	0.19	3.11	23.96
11	35	6	12	4.74	0.03	2.35	15.27
12	35	6	43	4.44	0.03	2.12	14.67
13	45	2	61	3.20	0.02	2.01	7.88
14	52	6	43	3.80	0.01	1.99	9.19
15	35	13	43	4.32	0.03	2.11	14.61
16	18	6	43	7.80	0.17	2.85	18.96

The lycopene content of thermosonicated watermelon juice was dependent on processing variables (Table 2). A significant increase in lycopene was observed at lower processing variables (25 °C, 2 min and 24 µm); these processing parameters recorded a lycopene content of 8.10 mg/100 g compared to the 6.19 mg/100 g observed in the fresh sample. Rawson et al.^36^ observed a similar pattern during the thermosonication of watermelon juice. The increase in lycopene content at lower processing variables was probably due to the growth, formation and violent collapse of small bubbles in the liquid during sonication, resulting from the fluctuation in pressure—a phenomenon known as acoustic cavitation^35^. In addition, at lower processing variables there are not enough violent cavitation bubbles due to low viscosity, and there are no dampening effects on collapse by high vapor pressure due to low temperature^37,38^.

**Figure 1:**
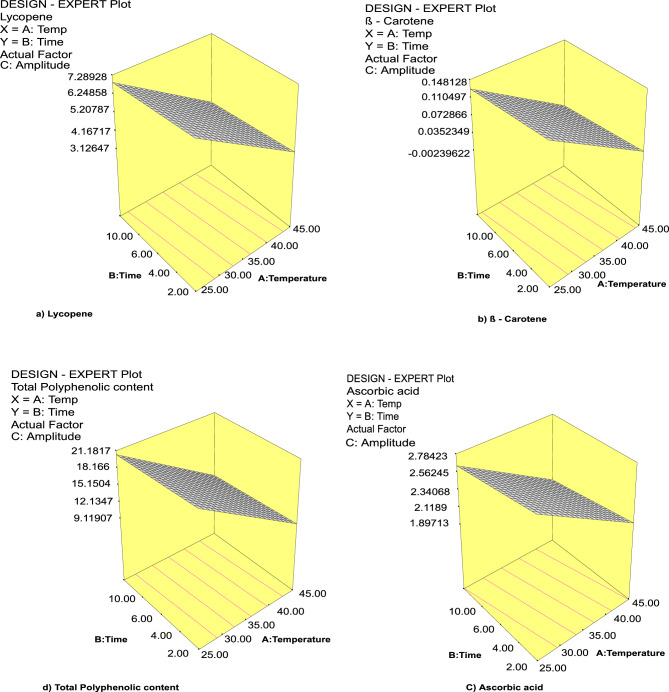
Response surface plots in terms of actual factor for phytochemicals in watermelon juice.

The analysis of variance showed a linear model and a significant lack of fit (*p* < 0.0001), indicating that the model was adequate (Table 3). The relation between independent variables and lycopene content was exhibited with a high correlation coefficient (R^2^ = 0.8378). A correlation coefficient close to 1 indicates the effectiveness of the correlation between predicted values and actual ones^31^. The adjusted determination coefficients (Adj R^2^) were also close to 1 (0.7973), which indicated that the experimental values could be significantly predicted by the model. The increase of lycopene in the thermosonicated samples, which was dependent on experimental conditions, decreased as processing variables increased. The response surface plots (Fig. 1a) showed a continuous decrease in lycopene content as processing variables increased.

**Table 3:** ANOVA for the linear model for watermelon phytochemicals.

Phytochemical	Source	Sum of squares	mean square	F value	*P* value	Remarks
Lycopene	Model significant	48.88	16.29	20.67	< 0.0001	Significant
	A	48.35	48.35	61.33	< 0.0001	
	B	0.43	0.43	0.54	0.4769	
	C	0.11	0.11	0.14	0.7189	
	Lack of fit	9.46	0.79	2832.77	< 0.0001	Significant
β-carotene	Model significant	0.060	0.020	36.16	0.0006	Significant
	A	0.059	0.059	0.58	< 0.0001	
	B	468E − 004	9.468E − 004	0.040	0.4616	
	C	6.590E − 005	6.590E − 005		0.8443	
	Lack of fit	0.020				Not significant
AA	Model significant	2.02	0.67	16.68	0.0001	Significant
	A	1.89	1.89	46.70	< 0.0001	
	B	0.055	0.055	1.36	0.2661	
	C	0.080	0.080	1.98	0.1852	
	Lack of fit	0.48				Not significant
TPC	Model significant	443.52	147.84	22.27	< 0.0001	Significant
	A	441.97	441.97	66.56	< 0.0001	
	B	1.25	1.25	0.19	0.6721	
	C	0.30	0.30	0.046	0.8342	
	Lack of fit	79.62	7.96	265.39	0.0038	Significant

A = Temperature; B = Time; C = Amplitude; *AA* ascorbic acid; *TCP* total polyphenolic content.

The mathematical equation in terms of coded factors showing effects on lycopene is shown in Eq. (1), where A is temperature, B is time and C is amplitude.

1 $${\text{Lycopene}} = { } + 12.29982 - 0.18816{\text{ X A}} - 0.049943{\text{ X B}} - 4.83786{\text{E}} - 003{\text{ X C }}$$

Shi and Maguer^39^ observed a similar trend during the thermosonication of tomato juice, with their study reporting that the lycopene content decreased with the combination of higher time–temperature conditions on tomato juice. This is because at high temperatures, the intensity of bubble collapse is weakened by the higher vapor pressure ^38^. Moreover, isomerization of *trans-*lycopene to the *cis* form, induced by heat and ultrasound during sonication treatment, leads to an undesirable decrease in lycopene content^4,26^. In addition, Khongthaw et al.^40^ reported that, the loss or decrease of lycopene is intensified during processing because lycopene can simply oxidize and rearrange into a cis isomer. The decrease may also be influenced by an oxidative injury occurring during thermosonication^41^. Moreover, the decrease in lycopene might be due to exposure to light during the experiment, since lycopene is an unstable phytochemical which isomerizes under light irradiation, high-temperature treatments and oxidation^42^.

### Effects of thermosonication on the β-carotene content of the watermelon juice

β-carotene is a phytochemical that has received considerable attention for being a precursor of vitamin A and shows strong biological activity as an antioxidant^43^. Fresh watermelon juice recorded 0.23 mg/100 g of β-carotene. The values of β-carotene content in the thermosonicated juice varied between 0.01 and 0.19 mg/100 g (Table 2). This study observed a decrease in β-carotene as processing variables were intensified, however, the amount of β-carotene was retained at lower processing variables (25 °C, 2 min and 24 µm), rather than at higher processing variables (Table 2). A similar trend was observed by other researchers who investigated the effects of thermosonication on fruit juices. Urango et al.^24^ reported that high temperatures during thermosonication were associated with an alteration of the food quality, and nutritional and sensory properties. In addition, the decrease in β-carotene content could be due to the cell disruption occurring during thermosonication processing^44^. During sonication, bubbles are released in the liquid medium, leading to an increase in enzyme activity which results in the formation of free radicals^45^. The rate of bubbles released at lower processing variables was found to be slower than those released at higher processing variables, while longer processing variables released bubbles at a faster rate and caused more cell disruption, which resulted in a gradual decrease of β-carotene^39^. The ANOVA results for β-carotene are shown in Table 3. The *p*-values less than 0.001 specify that the model was significant (Table 3). The values of determination coefficients (R^2^ = 0.7540) and adjusted determination coefficients (Adj. R^2^) were close to 1, which indicates a high degree of correlation between the experimental and predicted values. Each output variable in RSM has a mathematical relationship with the experimental variables by a linear equation. The equation for β-carotene can be expressed as follows: high temperatures can affect nutritional and sensory properties altering food quality.

2 $${\upbeta } - {\text{carotene}} = { } + 0.32255 - 6.58341{\text{E}} - 003{\text{ X A}} - 2.35703{\text{E}} - 003{\text{ X B}} - 1.20038{\text{E}} - 004{\text{X C}}$$

The generated response surface plot corresponding to each response showed the interactive effects of the variables (Fig. 1b). The loss of β-carotene was associated with high processing variables—as processing variables accelerated, the loss was intensified. Fonteles et al.^45^ reported that, under high processing variables the bubbles can disrupt the propagation of ultrasound waves, resulting in the loss of vital compounds such as β-carotene. Furthermore, Oberoi and Sogi^18^ reported that β-carotene content was decreased by acoustic cavitation (i.e., repeated cycles of compression and decompression produced during sonication). The acoustic cavitation may collapse less violently, which accelerates the decrease of β-carotene^39^. Acoustic cavitation leads to damage to the cellular structure of compounds, therefore natural liquid layers close the phase boundaries and consequently accelerate the process of mass transfer^45^. The development of free radicals and peroxide compounds produced during ultrasound at higher processing variables leads to a decrease in β-carotene^46^. Fonteles et al.^45^ further reported that the quantity of β-carotene was decreased by the free radicals which degraded the hydroxyl (–OH). This was due to the opening of the rings and the development of chalcone, which was caused by the temperature increase occurring during sonication^47^. High pressure and heat treatment were found to accelerate the isomerization of β-carotene^48^.

### Effects of thermosonication on the AA content of the watermelon juice

AA is an easily oxidized strong antioxidant compound which is naturally occurring and mostly found in plants species and their by-products^49^. It plays an important role in collagen tissue synthesis and the development of the immune system, and in reducing the risk of cardiovascular diseases and nervous disorders^50^. It is considered an indicator of quality in fruit juices^51^. For these reasons, it is crucial to keep AA stable and retain it during juice processing. The fresh watermelon juice recorded 3.47 mg/100 ml of AA (Table 2). This study observed a decrease in AA as processing variables were intensified, however, thermosonication retained about 3.11 mg/100 g of AA at lower processing variables (25 °C, 2 min and 24 µm). There was a constant decrease in AA with increasing temperature, time and amplitude (Table 2). According to Abdullah and Chin^51^, AA is not a stable compound therefore it is easily oxidised under extreme heat treatment. Moreover, Anaya-Esparza et al.^50^ reported that AA degradation is dependent on amplitude, acoustic energy density, temperature, and treatment time during thermosonication. Tiwari et al.^52^ reported 5% decrease in AA in orange juice during thermosonication; while Adekunte et al.^53^ observed a 32% decrease in tomato juice during thermosonication. The decrease in AA at higher processing variables might be attributed to severe physical conditions occurring as a result of the cavitation collapse of bubbles during sonication treatment^28^. In addition, the decrease in the AA content may be attributed to cavities (formed by sonication) which may be filled with water vapor and gases dissolved in the juice, such as O_2_ and N_2_^45^. Therefore, moderate processing temperature is needed to ensure higher retention of ascorbic acid as it is a highly heat sensitive compound. The analysis of variance showed a significant linear model and a non-significant lack of fit (Table 3). The Predicted R^2^ (0.81) was in reasonable agreement with the Adjusted R^2^ (0.76). The regression equation of the model is presented in Eq. (3), where A is temperature, B is time and C is amplitude.

3 $${\text{Ascorbic acid}} = { } + 3.92780 - 0.037171{\text{ X A}} - 0.017961{\text{ X B}} - 4.17767{\text{E}} - 003{\text{ X C}}$$

The plot shown in Fig. 1c illustrates the combined effects of temperature, time and amplitude on AA. The decrease was observed to be intensified at high processing variables (Table 2). Rawson et al.^36^ reported that AA is an unstable compound which degrades easily at higher processing variables. The decrease may also be due to the presence of oxygen and the breaking down of molecules during sonication^53^. Rawson et al.^36^ reported that the formation of hydroxyl radicals by water during sonication, which may occur at the gas–liquid interface causing oxidation and several sonochemical reactions to occur simultaneously, may lead to a decrease in AA. Tiwari et al.^52^ reported that AA content decreased due to a decrease in other compounds, since it has a protective effect on other compounds such as phenols. Sonication can result in enhanced free radicals, which leads to the oxidation of AA content, even though it induces degassing by cavitation of air-saturated systems^44^. A decrease in AA is associated with a decrease in color and pH, since it acts as a valid criterion for natural pigments and aromatic substances^42^. Thermosonication was found to retain the AA even at high processing variables. Therefore, thermosonication is a recommended processing method for retaining the considerable content of AA.

### Effects of thermosonication on the TPC of the watermelon juice

TPC have protective effects on human health due to their strong antioxidant properties which combat free radicals in the body^50^. The results revealed a TPC of 25.95 mg GAE/g (Table 2) in fresh watermelon juice, while the TPC of thermosonicated watermelon juice varied between 7.88 and 23.96 mg GAE/g (Table 2). A decrease in TPC was observed in thermosonicated watermelon juice, which was more significant with increasing thermosonication intensity. Thermosonication retained about 23.96 mg/GAE/g at lower processing variables (25 °C, 2 min and 24 µm). However, TPC was retained even at the highest processing variables (Table 2). These observations align with those reported by Rawson et al.^36^ [whose study found that thermosonication retained the TPC in watermelon juice]. The decrease in TPC content could be due to high-energy cavitation bubbles containing solvent vapor^44^. These bubbles implode near cell walls, causing very high local temperatures, pressure increase and cell wall destruction, which ease mass transfer from cell to solvent, and enhance the loss of important phytochemicals^42^. The bubbles accelerate chemical reactions, increase diffusion rates, disperse aggregates, or inactivate enzymes and microorganisms^41,48^. Cavitational bubbles filled with water vapor and O_2_ gas dissolved in the juice during thermosonication, resulting in decreased TPC^28,36^. TPC is highly sensitive to heat, therefore a temperature increase accelerated the decrease of TPC^54^. Linear effects (temperature, time and amplitude) and their interaction were found to be significant, showing a 95% confidence interval (R^2^ = 0.8477) from a low p-value (*p* < 0.0001) and a high F-value (Table 3). The F-value of 22.27 implies that the model was significant (Table 3). Notably, there is only a 0.01% chance that an F-value this large could occur due to noise. The TPC of fresh juice was decreased by increasing the thermosonication variables (Fig. 1d). However, the decrease in TPC was not severe at lower processing variables (Fig. 1d).

Equation (4) illustrates the relationship between temperature, time and amplitude:

4 $${\text{Total polyphenolic content }} = { } + 35.92301 - 0.56888{\text{ X A}} - 0.085627{\text{ X B}} - 8.1516{\text{E}} - 0.003{\text{ X C}}$$

There was an increase in viscosity and a weak intensity of bubble collapse at high heat and vapor pressure, which resulted in a polyphenol decrease^53^. The collapse of the bubbles during sonication caused shock waves to pass through the solvent, enhancing the mass transfer within the system^42^, which led to the destruction of phytochemicals.

### Numerical optimization and model fitting of experimental conditions

Optimization was carried out to determine the precise and optimal parameter combinations which retained the quality of parameters of the watermelon juice^29^. Temperature, time and amplitude were independent variables while lycopene, β-carotene, AA and the TPC of the watermelon juice were dependent variables. The ANOVA showed that the resultant linear models adequately represented the experimental data for the phytochemicals under investigation. The predicted values were closely correlated with the experimental data, as demonstrated by the R^2^. The *p*-values and f-values of the model were < 0.0001 for lycopene, β-carotene, AA, and TPC, which confirmed the goodness of fit of the model (Table 3). The optimum processing variables that retained the quality parameters were 25 °C for 2 min at an amplitude of 24 µm at a constant frequency of 25 kHz. These conditions resulted in lycopene of 8.10 mg/100 g, β-carotene of 0.19 mg/100 g, AA of 3.11 mg/100 g and TPC of 23.96 mg/GAE/g with a desirability of 0.81.

## Conclusion

The perishability and non-thermal nature of watermelon juice called for the application of non-thermal innovative technologies to extend the shelf-life thereof and expand its utilization. In this study, thermosonication was applied to process the watermelon juice, with a view to retaining its critical quality parameters (lycopene, β-carotene, AA and TPC) using the RSM. The highest quality parameters were retained at 25 °C, 2 min, 24 µm and a constant frequency of 25 kHz. This study observed an increase in lycopene at lower processing variables and a decrease as processing variables intensified. A decrease in β-carotene, AA and TPC was also observed as processing variables intensified, however, it was proven that thermosonication retained all the quality parameters which were investigated. Temperature and time were key factors in determining the decrease in quality parameters. All dependent variables were highly significant (*p* < 0.0001) to the independent variables studied, apart from the β-carotene which was non-significant (*p* > 0.05). The ANOVA results showed that the proposed model was adequate (*p* < 0.0001), significant, and fit all quality parameters. The R^2^ for predicted quality parameters showed a good correlation with the experimental data at a confidence level of 95%. The results revealed the advantages of thermosonication for watermelon juice processing. At low processing variables, thermosonication enhanced and retained the phytochemicals of the watermelon juice. Although the application of thermosonication in beverage-processing industries is still not common, the findings indicate that such processing could be a worthwhile potential technique to produce watermelon juice with enhanced overall quality. The exploration into its effect on microbial activity to better the shelf-life extension as well as on consumer preferences could enhance the market to the diversification of the beverage industry.

## Data Availability

The datasets used and/or analysed during the current study available from the corresponding author on reasonable request, request from the corresponding author at maotomossa@gmail.com.
