# Author Correction: Optimization of thermosonication conditions for critical quality parameters of watermelon juice using response surface methodology

**DOI:** 10.1038/s41598-025-88786-8

**Published:** 2025-02-13

**Authors:** Makaepea M. Maoto, Daniso Beswa, Afam I. O. Jideani

**Affiliations:** 1 Department of Food Science and Technology, Faculty of Science, Engineering and Agriculture, University of Venda, Private Bag 5050, Thohoyandou, Limpopo 0950 South Africa; 2 Department of Life and Consumer Sciences, School of Agriculture and Life Sciences, College of Agriculture and Environmental Sciences, University of South Africa, Private Bag x6, Florida, Gauteng 1710 South Africa; 3 Special Interest Group Post Harvest Handling, ISEKI-Food Association, Muthgasse 18, 1190 Vienna, Austria; 4 University of Johannesburg, Johannesburg, South Africa

Correction to: *Scientific Reports* 10.1038/s41598-024-64066-9, published online 14 June 2024

Daniso Beswa was omitted from the author list in the original version of this Article.

The Author Contributions section now reads:

“Ms M.M.M was the main researcher. Dr D.B and Prof A.I.O.J. were the co-supervisor and main supervisor, respectively.”

The original Article has been corrected.

